# Myeloid-Specific Deletion of the AMPKα2 Subunit Alters Monocyte Protein Expression and Atherogenesis

**DOI:** 10.3390/ijms20123005

**Published:** 2019-06-19

**Authors:** Beate Fisslthaler, Nina Zippel, Randa Abdel Malik, Fredy Delgado Lagos, Sven Zukunft, Janina Thoele, Daniel Siuda, Oliver Soehnlein, Ilka Wittig, Juliana Heidler, Andreas Weigert, Ingrid Fleming

**Affiliations:** 1 Institute for Vascular Signalling, Centre for Molecular Medicine, Goethe University, 60596 Frankfurt am Main, Germany; NZippel@gmx.de (N.Z.); AbdelMalik@vrc.uni-frankfurt.de (R.A.M.); Lagos@vrc.uni-frankfurt.de (F.D.L.); zukunft@vrc.uni-frankfurt.de (S.Z.); janinathoele@gmx.de (J.T.); Siuda@vrc.uni-frankfurt.de (D.S.); fleming@em.uni-frankfurt.de (I.F.); 2 German Center of Cardiovascular Research (DZHK), Partner site RhineMain, 60596 Frankfurt am Main, Germany; wittig@med.uni-frankfurt.de (I.W.); julianaheidler@googlemail.com (J.H.); 3 Institute for Cardiovascular Prevention, Ludwig-Maximilians-University, 80336 Munich, Germany; oliver.soehnlein@gmail.com; 4 German Center for Cardiovascular Research (DZHK), Partner Site Munich Heart Alliance, 80336 Munich, Germany; 5 Department of Physiology and Pharmacology and Department of Medicine, Karolinska Institute, 17177 Stockholm, Sweden; 6 Functional Proteomics, SFB 815 Core Unit, Goethe University, 60596 Frankfurt am Main, Germany; 7 Institute of Biochemistry I, Goethe-University Frankfurt, 60596 Frankfurt am Main, Germany; weigert@biochem.uni-frankfurt.de

**Keywords:** DNA methylation, matrix metalloproteinase, macrophage

## Abstract

The AMP-activated protein kinase (AMPK) is an energy sensing kinase that is activated by a drop in cellular ATP levels. Although several studies have addressed the role of the AMPKα1 subunit in monocytes and macrophages, little is known about the α2 subunit. The aim of this study was to assess the consequences of AMPKα2 deletion on protein expression in monocytes/macrophages, as well as on atherogenesis. A proteomics approach was applied to bone marrow derived monocytes from wild-type mice versus mice specifically lacking AMPKα2 in myeloid cells (AMPKα2^∆MC^ mice). This revealed differentially expressed proteins, including methyltransferases. Indeed, AMPKα2 deletion in macrophages increased the ratio of S-adenosyl methionine to S-adenosyl homocysteine and increased global DNA cytosine methylation. Also, methylation of the vascular endothelial growth factor and matrix metalloproteinase-9 (MMP9) genes was increased in macrophages from AMPKα2^∆MC^ mice, and correlated with their decreased expression. To link these findings with an in vivo phenotype, AMPKα2^∆MC^ mice were crossed onto the ApoE^-/-^ background and fed a western diet. ApoExAMPKα2^∆MC^ mice developed smaller atherosclerotic plaques than their ApoExα2^fl/fl^ littermates, that contained fewer macrophages and less MMP9 than plaques from ApoExα2^fl/fl^ littermates. These results indicate that the AMPKα2 subunit in myeloid cells influences DNA methylation and thus protein expression and contributes to the development of atherosclerotic plaques.

## 1. Introduction

The AMP-activated protein kinase (AMPK) is a heterotrimeric serine/threonine kinase consisting of various combinations of α, β, and γ subunits that are differentially expressed in different cells and tissues. The AMPK is one of the major sensors of energy status in mammalian cells and as such plays an essential role in the regulation of cellular homeostasis, metabolism, cell growth, differentiation, apoptosis, and autophagy (for review see [1,2]). The sequences of the human AMPKα1 and α2 isoforms are 90% identical and most cell types express both isoforms. Despite this, the specific deletion of the isoforms results in very distinct phenotypes. For example, while AMPKα1^-/-^ mice suffer from severe anemia [3], as a consequence of alteration in erythrocyte membrane deformability [4], AMPKα2^-/-^ mice are insulin-resistant and glucose-intolerant [5,6]. The subcellular localization of the two isoforms may at least partially explain their apparent distinct actions, as the AMPKα2 subunit seems to be more frequently enriched in the nucleus than the AMPKα1 subunit [1].

The AMPK also has an important role in regulating vascular function, with distinct roles reported in endothelial cells, vascular smooth muscle cells, and immune cells (for review see [7]). While AMPKα1 accounts for most of the AMPK activity in endothelial cells [8,9,10,11], specific downregulation of the α2 isoform has clear effects on cell function [12,13]. A lot less is known about the role of the AMPKα subunits in the regulation of myeloid cell function. One possible reason for the apparent lack of interest in the role of AMPK in myeloid cells may be related to its low expression in freshly isolated monocytes. However, the expression of the AMPKα1 subunit is reported to increase markedly following the initiation of monocyte differentiation into macrophages [14], and it now seems that the predominant role of the kinase is in regulating the inflammatory potential of macrophages. In mice, AMPKα1 activation has been linked with the suppression of pro-inflammatory responses and the promotion of monocyte differentiation to a phenotype that promotes the resolution of inflammation [14,15,16]. This fits well with the report that AMPKα1 activation is linked to the expression of the angiotensin-converting enzyme in human macrophages and the decreased generation of pro-inflammatory cytokines [17]. Almost nothing is known about the role of the AMPKα2 in myeloid cells, which is expressed at much lower levels than the α1 isoform [15]. However, the AMPKα2 subunit was reported to play a determinant role in neutrophil survival in hypoxic conditions in ischemic hindlimbs and thereby contributes to the processes of arteriogenesis and angiogenesis [18]. Given this information, the aim of the current investigation was to determine whether the myeloid specific deletion of the AMPKα2 subunit had any effect on the function of monocytes and/or monocyte-derived macrophages that could be linked to the development of atherosclerosis.

## 2. Results

### 2.1. Consequences of the Myeloid-Specific Deletion of AMPKα2 on Monocyte Protein Expression

**Figure 1:**
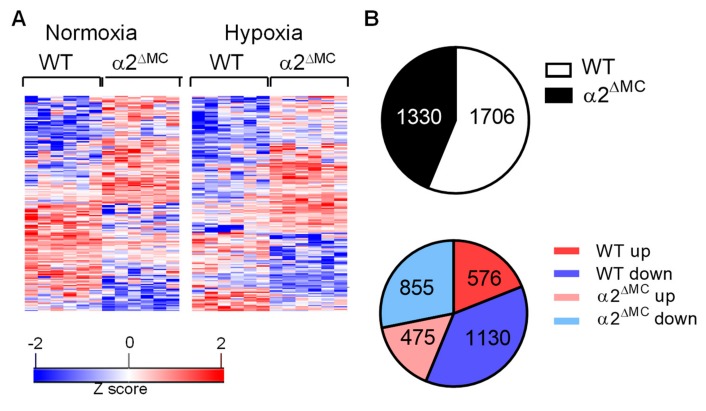
Differentially expressed proteins in monocytes from wild-type and AMP-activated protein kinase (AMPK)α2^∆MC^ littermates. Bone marrow monocytes were isolated and maintained under normoxic or hypoxic conditions for 16 h. (**A**) Heat map showing differential protein expression in monocytes from wild-type (WT) and AMPKα2^∆MC^ (α2^∆MC^) mice. Adherent cells were cultured for 16 h in normoxic (left) or hypoxic conditions (right) and total proteins were subjected to digestion with trypsin and proteomics; *n* = 6 mice per group. (**B**) Proteins differentially regulated by hypoxia in monocytes from wild-type (WT) and AMPKα2^∆MC^ (α2^∆MC^) mice; upper panel: total numbers of differentially regulated proteins, (lower panel) up- and down-regulated proteins.

To determine the consequences of AMPKα2 deletion on monocyte function, proteomic analyses of bone-marrow-derived monocytes was performed using bone marrow-derived monocytes from wild-type and AMPKα2^∆MC^ littermates. Experiments were performed under basal conditions, as well as in response to hypoxia (1% O_2_, 16 h) to ensure AMPK activation. The wild-type and AMPK α2^∆MC^ samples clustered according to the genotype (Figure 1A, Supplementary Table S1). In monocytes from wild-type mice, 1706 proteins were differentially regulated by hypoxia; 1130 down regulated and 576 up regulated. In the monocytes from AMPKα2^∆MC^ mice, hypoxia altered the expression of 1330 proteins; 855 down- and 475 up-regulated (Figure 1B). 

### 2.2. Consequences of Myeloid Cell AMPKα2 Deletion on the S-adenosyl Methionine Metabolism 

**Figure 2:**
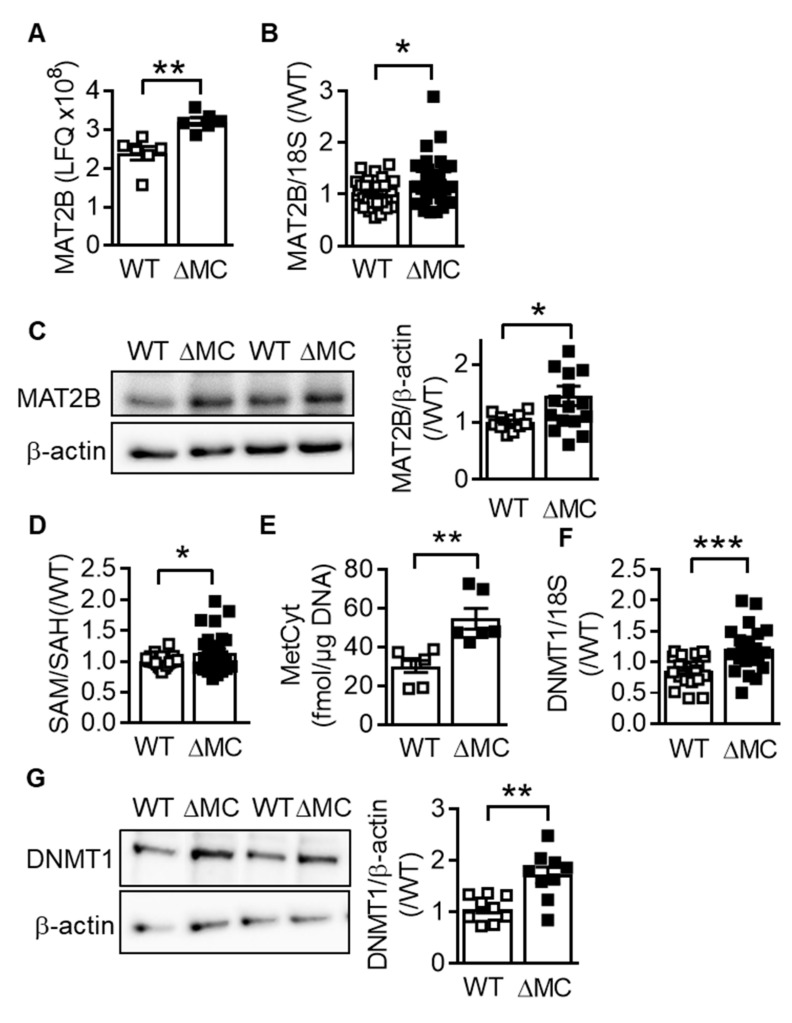
Consequences of myeloid cell AMPKα2 deletion on the S-adenosyl methionine metabolism. (**A**) Results of the proteomics experiments using monocytes from wild-type (WT) and AMPKα2^∆MC^ (∆MC) mice exposed to hypoxia (1% O_2_; 16 h); *n* = 6 animals per group. (**B**) MAT2B mRNA expression in macrophages from wild-type (WT) and AMPKα2^∆MC^ (∆MC) mice; *n* = 16 animals per group (each sample in duplicate). (**C**) MAT2B protein expression in macrophages from wild-type (WT) and AMPKα2^∆MC^ (∆MC) mice; *n* = 13–16 animals per group. (**D**) SAM/SAH ratio in macrophages from wild-type (WT) and AMPKα2^∆MC^ (∆MC) mice; *n* = 13 animals per group. (**E**) Methyl cytosine levels in total genomic DNA from macrophages from wild-type (WT) and AMPKα2^∆MC^ (∆MC) mice; *n* = 6 animals per group. (**F**) DNMT1 RNA expression, *n* = 11 animals per group (each sample in duplicate) and (**G**) protein expression, *n* = 10 animals per group. (Students *t*-test) * *p* < 0.05, ** *p* < 0.01, *** *p* < 0.001.

Among the proteins significantly upregulated in monocytes from AMPKα2^∆MC^ mice was methionine adenosyltransferase (MAT) 2B (Figure 2A) the catalytic subunit of the enzyme that catalyzes the conversion of methionine to S-adenosyl methionine (SAM) [19]. The increased expression of MAT2B could be confirmed at the mRNA (Figure 2B) and protein levels (Figure 2C), and fitting with the altered protein expression, the ratio of SAM to S-adenosyl homocysteine (SAH) was increased in the AMPKα2–deficient macrophages (Figure 2D). During the conversion of SAM to SAH, methyl groups generated for the methylation processes and global DNA cytosine methylation were higher in the AMPKα2^∆MC^ than in wild-type cells (Figure 2E). AMPKα2 deletion also increased the expression of DNA methyltransferase (DNMT)1, which catalyzes DNA methylation to limit transcription factor access to promoters (Figure 2F,G).

### 2.3. Consequences of Myeloid Cell AMPKα2 Deletion on DNA Methylation

**Figure 3:**
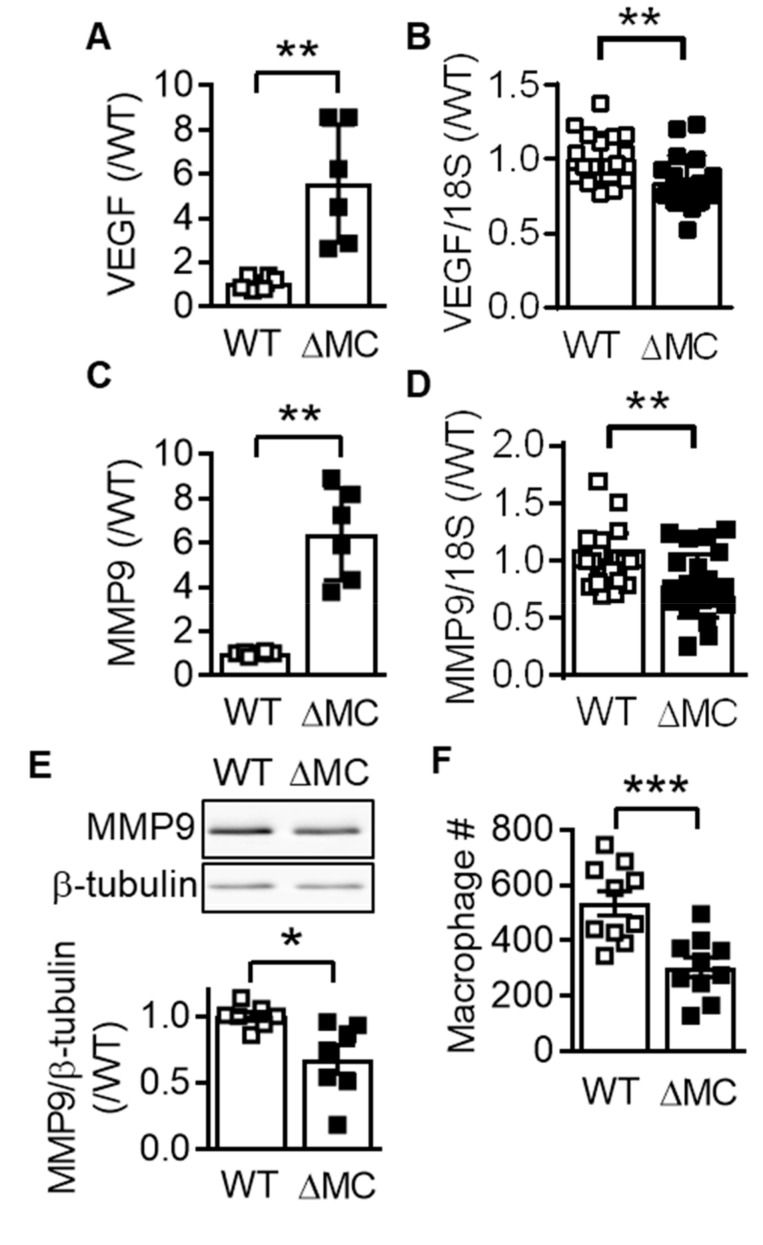
Consequence of myeloid AMPKα2 deletion on the expression of vascular endothelial cell growth factor (VEGF) and MMP9. (**A**) *Vegfa* DNA in methylcytosine immunoprecipitates from genomic DNA isolated from wild-type (WT) and AMPKα2^∆MC^ (∆MC) macrophages (*n* = 6). (**B**) VEGF mRNA expression in bone marrow-derived macrophages from wild-type (WT) and AMPKα2^∆MC^ (∆MC) mice (*n* = 10 each in duplicate). (**C**) *Mmp9* DNA in methylcytosine immunoprecipitates from genomic DNA isolated from wild-type (WT) and AMPKα2^∆MC^ (∆MC) macrophages (*n* = 6). (**D**) MMP9 mRNA expression in bone marrow-derived macrophages from wild-type (WT) and AMPKα2^∆MC^ (∆MC) mice (*n* = 10 each in duplicate). (**E**) MMP9 protein levels in monocytes from wild-type (WT) and AMPKα2^∆MC^ (∆MC) mice (*n* = 13, each in duplicate). (**F**) Number of wild-type (WT) and AMPKα2^∆MC^ (∆MC) macrophages that migrated through fibronectin; *n* = 6, each performed in duplicate (Student’s *t*-test). * *p* < 0.05, ** *p* < 0.01, *** *p* < 0.001.

The methylation of cytosine residues in cytosine-guanine dinucleotide (CpG) islands play a major role in chromatin compaction and consequently gene silencing [19,20]. Given that the expression of vascular endothelial cell growth factor (VEGF) and matrix metalloproteinase (MMP) 9 are known to be regulated by methylation [21,22], the methylation of CpG islands was assessed in macrophages from wild-type and AMPKα2^∆MC^ mice. The methylation of the CpG island, which overlaps the 5′untranslated region and the first exon, in the *Vegfa* gene, was higher in macrophages from AMPKα2^∆MC^ than from wild-type mice (Figure 3A), a phenomenon that correlated with a decrease in VEGF expression (Figure 3B). Similarly, methylation of the *Mmp9* CpG island, which is intragenic and spans from exon 3 to exon 4, was also higher in AMPKα2-deficient than in AMPKα2-expressing macrophages (Figure 3C), and correlated with a decrease in MMP9 mRNA in monocytes (Figure 3D) and protein levels (Figure 3E). Functionally, these changes were reflected by a decreased ability of AMPKα2^∆MC^ macrophages to invade fibronectin-coated filters compared to macrophages from wild-type mice (Figure 3F). 

### 2.4. Consequence of AMPKα2 Deletion on Macrophage Polarization

**Figure 4:**
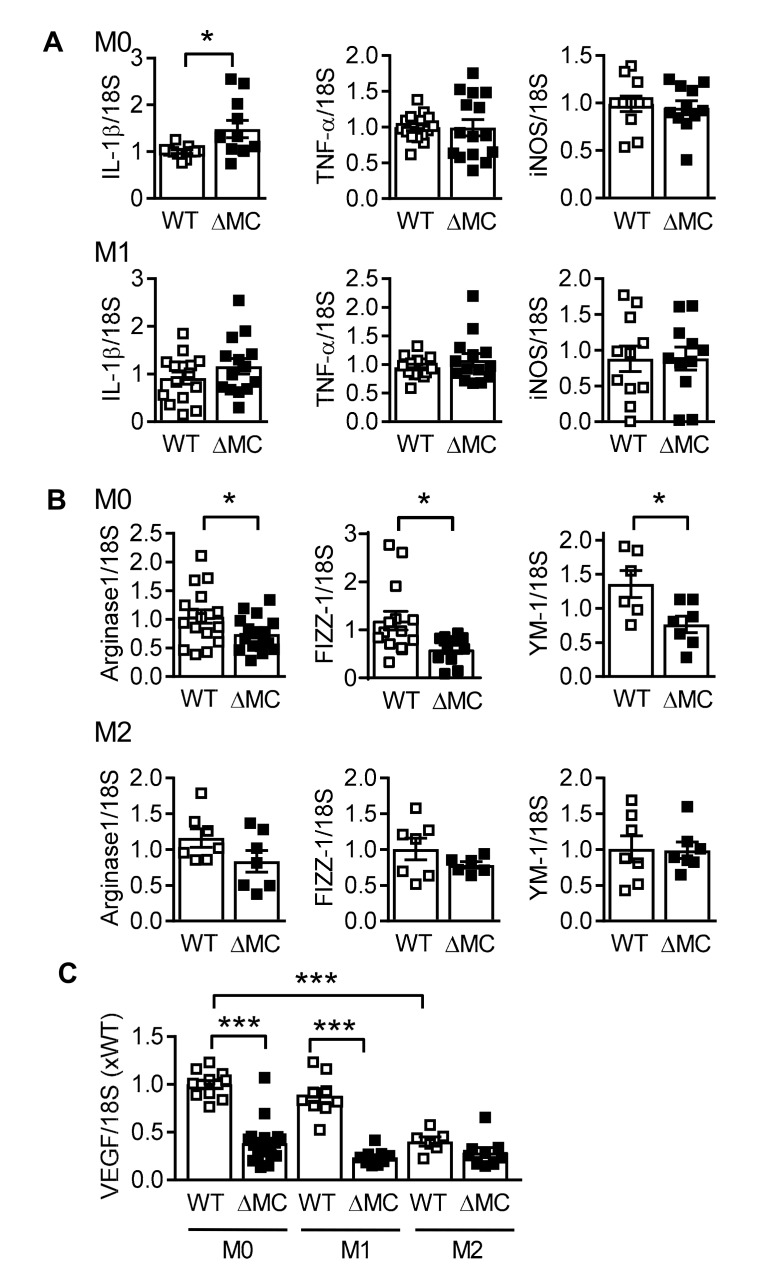
Consequence of AMPKα2 deletion on macrophage polarization. Expression (mRNA) of polarization markers in bone marrow-derived macrophages from wild-type (WT) and AMPKα2^∆MC^ (∆MC) mice under basal conditions (M0) or after polarization with (**A**) LPS (0.1 ng/mL) and IFN-γ (1 ng/mL), or (**B**) IL-4 (1 ng/mL) and IL-13 (1 ng/mL); *n* = 6–9 animals per group, each sample determined in duplicate (Student’s *t*-test). (**C**) VEGF mRNA expression in bone marrow-derived macrophages from wild-type (WT) and AMPKα2^∆MC^ (∆MC) mice under basal conditions (M0) or after polarization with LPS and IFN-γ (M1), or IL-4 and IL-13 (M2); *n* = 7 to 10 animals per group, each sample determined in duplicate (Students *t*-test). * *p* < 0.05, *** *p* < 0.001.

Next, we assessed the expression of classical macrophage activation markers under basal conditions and following polarization with either the combination of lipopolysaccharide and interferon (IFN)-γ for 4 h (M1 polarization) or interleukin (IL)-4 and IL-13 for 16 h (M2 polarization). Differences in the expression of IL-1β, resistin like beta (FIZZ1, found in inflammatory zone) and chitinase-like 3 (YM1) were evident in macrophages from wild-type and AMPKα2^∆MC^ mice under basal conditions (Figure 4A,B). While the polarization protocol increased the expression of IL-1β by 2000 fold, FIZZ1 by 5000 fold, and arginase by 18 fold, there were no differences between the cells from wild-type and AMPKα2^∆MC^ mice. Macrophage polarization also affects the expression of VEGF and, consistent with the previous results, VEGF expression was attenuated in macrophages from AMPKα2^∆MC^ mice under basal conditions, as well as following cell stimulation with bacterial lipopolysaccharide (LPS) and IFN-γ (Figure 4C). M2 polarization decreased VEGF expression in macrophages from wild-type mice, but did not induce a further decrease in cells from AMPKα2^∆MC^ mice.

### 2.5. Effect of AMPKα2 Deletion in Myeloid Cells on Atherosclerotic Plaque Formation

**Figure 5:**
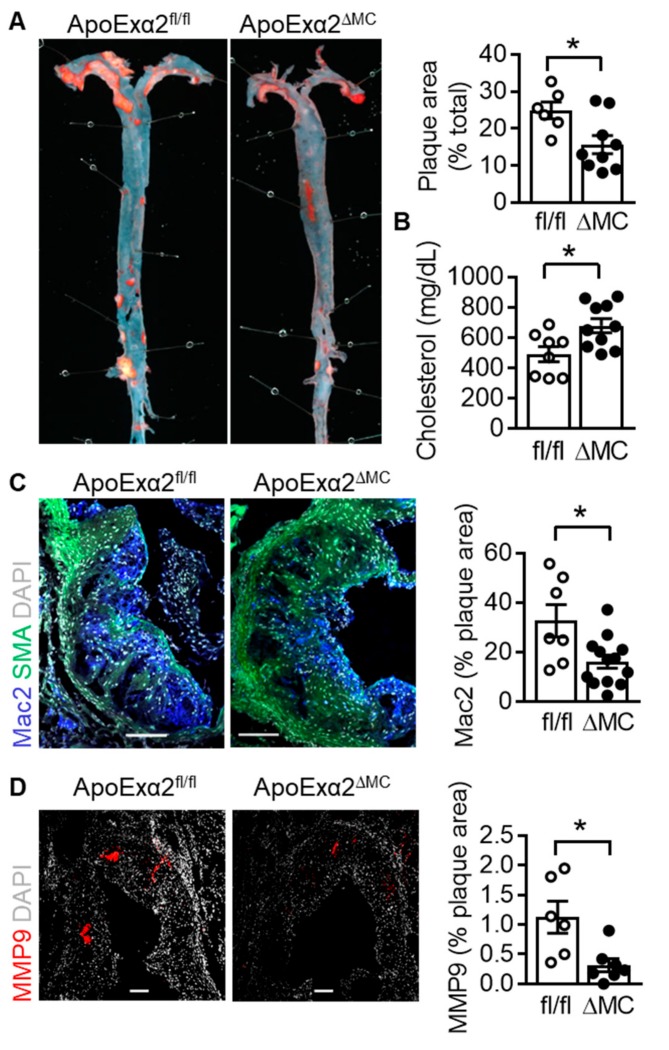
Effect of AMPKα2 deletion in myeloid cells on atherosclerotic plaque formation. ApoExα2^fl/fl^ (fl/fl) and ApoExα2^∆MC^ (∆MC) mice were fed a western diet for four months. (**A**) Representative pictures of oil red O stained dissected aortae and quantification of the plaque area; *n* = 7 to 10 animals per group (Student’s *t* test). (**B**) Cholesterol levels in serum from ApoExα2^fl/fl^ (fl/fl) and ApoExα2^∆MC^ (∆MC) fed a western diet for four months; *n* = 8 to 10 animals per group (Student’s *t*-test). (**C**) Mac2 (blue) and smooth muscle actin (green) expression in aortic root plaques from ApoExα2^fl/fl^ (fl/fl) and ApoExα2^∆MC^ (∆MC) mice, DAPI = grey; plaques from *n* = 7–10 mice per group. Size bar = 100 µm (Student’s t test). (**D**) MMP9 (red) expression in aortic root plaques from ApoE^-/-^ (fl/fl) and ApoExα2^∆MC^ (∆MC) mice, DAPI = grey; *n* = 5 to 6 animals per group. Size bar = 100 µm (Student’s *t*-test). * *p* < 0.05.

Monocyte infiltration into the vascular wall and their differentiation to macrophages is one of the key events in atherogenesis. Therefore, to assess the consequences of AMPKα2 deletion in monocytes/macrophages on the latter process, AMPKα2^∆MC^ mice were crossed onto the apolipoprotein E–deficient (ApoE^-/-^) background to generate ApoExα2^∆MC^ mice and fed a western diet for four months. The western diet elicited the deposition of atherosclerotic plaques in the aortic root as well as in the aortae of ApoExα2^fl/fl^ mice (Figure 5A). A significantly smaller plaque area was detected in aortae from ApoExα2^∆MC^ mice. This was despite the fact that serum levels of cholesterol were actually higher in the ApoExα2^∆MC^ mice (Figure 5B), which is consistent with previous reports that the AMPK phosphorylates and inhibits the HMG CoA reductase [20,21]. 

Immunohistochemistry of the aortic root after four months of a western diet, however, revealed a clear decrease in macrophage infiltration (Mac2 positive regions) in plaques from the ApoExα2^∆MC^ mice (Figure 5C). This coincided with a decrease in MMP9 expression (Figure 5D) and an increase in plaque collagen levels (Supplementary Figure S1). However, although there was a trend towards a lower infiltration of neutrophils and monocytes in the ApoExα2^∆MC^, when the aortae were digested and the cell content assessed by FACS analyses (Supplementary Figure S2). It failed to achieve statistical significance, as even the total number of infiltrating CD45+ cells into the vascular wall was highly variable between the experimental groups. The acute adherence of monocytes to the carotid artery also showed a trend towards decreased attachment in the ApoExα2^∆MC,^ but again failed to achieve statistical significance (data not shown). 

**Figure 6:**
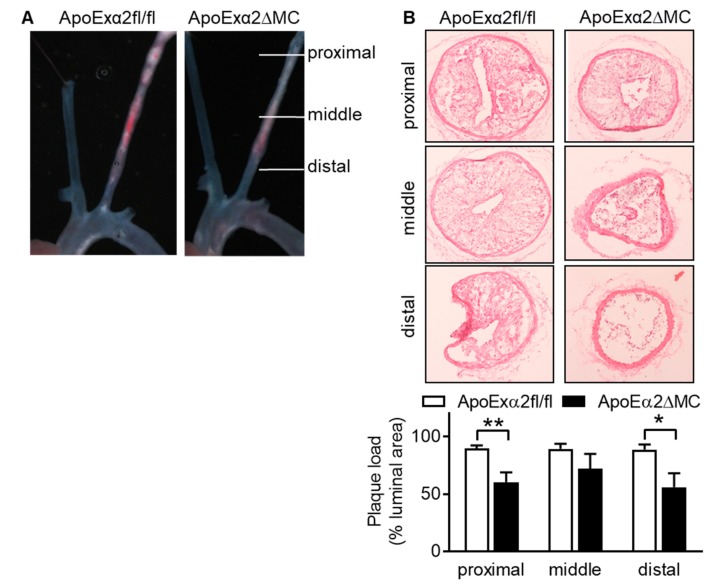
Effect of myeloid cell specific AMPKa2 deletion on plaque formation after partial carotid artery ligation. Three weeks after partial ligation, the left carotid artery in ApoExα2^fl/fl^ and ApoExα2^∆MC^ mice carotid arteries were dissected. (**A**) Representative images of plaques in the intact carotids. The images shown are representative on an additional four animals in each group. (**B**) Representative cross sections of the ligated carotid at different distances from the ligation site. Side bar = 100 µm. The bar graph summarizes the plaque load (% of total luminal area); *n* = 4–5 animals per group (Student’s *t* test). * *p* < 0.05, ** *p* < 0.01.

In an accelerated model of atherosclerosis linked to disturbed flow i.e., partial ligation of the carotid artery combined with a high fat diet over three weeks [22], the carotid arteries from the ApoExα2^∆MC^ mice also developed smaller plaques than carotid arteries from ApoExα2^ff/fl^ mice (Figure 6). 

## 3. Discussion

The results of the present study indicate that the deletion of the AMPKα2 subunit in myeloid cells significantly alters protein expression in bone marrow-derived monocytes. These changes correlated with altered gene expression and invasion capacityas well as a subtle change in macrophage polarization. When the AMPKα2^∆MC^ mice were crossed onto the ApoE^-/-^ background, the overall result was a small but consistent decrease in the atherosclerotic plaque formation in two different models.

One of the significantly regulated proteins was MAT2B, the catalytic subunit of the methionine adenosyltransferase that catalyzes the conversion of methionine to SAM. Consequently, there was an increase in the ratio of SAM to SAH in AMPKα2-deficient cells that was accompanied by an increase in global DNA cytosine methylation, reflecting the generation of methyl groups during the conversion of SAM to SAH. The expression of numerous genes has been linked to altered methylation and the methylation of CpG islands within *Vegfa* and *Mmp9* was increased in macrophages from AMPKα2^∆MC^ mice. Our data provide evidence for what has to-date only been a tentative link between MAT2B and VEGF; as increased serum levels of folate, a cofactor for SAM synthesis increased the methylation of the *Vegf* and monocyte chemoattractant protein-1 promoters resulting in a decrease in their expression and reduced atherosclerosis [23]. MAT2B was not the only methylation-regulating enzyme affected by AMPKα2 deletion in monocytes as DNMT1 expression also increased. AMPK has been reported by others to phosphorylate and inhibit DNMT1 in endothelial cells [24], thus our data suggest that the kinase can affect DNMT1-dependent methylation by at least two mechanisms i.e., by regulating its expression as well as its phosphorylation.

The AMPK is probably best known for its role in the regulation of energy metabolism but it is now clear that it also plays a role in inflammatory responses. This is of particular interest given the link between chronic low-grade inflammation in the cardiovascular system and metabolic dysregulation (for review see [25]). Despite current interest in the AMPK, not a lot is known about the role of the different AMPKα subunits in the regulation of myeloid cell function. There is consensus that the α1 subunit is the major AMPKα subunit in monocytes and macrophages and that it seems to regulate inflammatory signaling [14,15,16]. One determinant of monocyte/macrophage AMPKα1 expression seems to be a microRNA i.e., miR-33 [26], which has also been linked with metabolic regulation and atherosclerosis [27]. Limited information is available regarding the potential role of the AMPKα2 subunit in monocytes and macrophages but in the current study, the deletion of the AMPKα2 subunit was found to affect the expression of macrophage markers. However, marked changes were evident in unpolarized macrophages and not those differentiated to the classically activated (M1) or alternatively activated (M2) phenotypes. These observations suggest that strong inflammatory stimuli blunt the impact of AMPKα2 on macrophage polarization marker expression.

The downregulation of the AMPKα2, but not the AMPKα1 subunit was previously reported to attenuate neutrophil isocitrate dehydrogenase expression and the production of α-ketoglutarate [18]. As a consequence, AMPKα2 deletion altered the expression of hypoxia-inducible factor-1α as well as the genes regulated by it, and impacted on neutrophil survival. The defects in neutrophil survival that resulted from the deletion of the AMPKα2 subunit were linked with the expression of a network of proteins—many of which affect mitochondrial function and cell survival. In the absence of these mechanisms, the recruitment of pro-angiogenic monocytes to ischemic areas was also reduced [18]. Certainly in addition to the liberation of VEGF, MMPs and other pro-angiogenic growth factors [28], neutrophils also release factors such as cathelicidins that promote the recruitment of monocytes [29,30], which then amplify arteriogenic as well as atherogenic processes. Although the results of the current study focused on atherosclerosis, the observed decrease in monocyte recruitment to atherosclerotic plaques in the AMPKα2-deficient mice may well be linked to neutrophil loss. This implies that the same mechanism may underlie the decreased expression of proangiogenic factors in hindlimbs from AMPKα2^∆MC^ mice, as well as the decrease in atherosclerotic plaque formation. 

Few studies have assessed the consequences of AMPKα1 versus AMPKα2 deletion in a head-to-head manner. Recently, a direct comparison of the consequences of the global deletion of AMPKα1 and AMPKα2 on collateral remodeling and arteriogenesis revealed that the loss of both proteins attenuated the recovery of blood flow after ischemia [31]. The consequences of AMPKα1 deletion appeared more severe and were replicated in mice specifically lacking the AMPKα1 subunit in myeloid cells [31]. As such, the observations directly contradict a study published shortly thereafter, which directly compared the consequences of the myeloid cell–specific deletion of the AMPKα1 with that of the α2 subunit in the same model of hindlimb ischemia [18]. In the latter study, the consequences of AMPKα2 subunit deletion resulted in a more severe phenotype. The reasons for these contradictory findings are currently unclear; even though one study focused on macrophages and the other on neutrophils, both made use of the same LysM-cre deleter mice to target the myeloid lineage. The situation is equally confused with regards to atherosclerosis as AMPKα2 deletion (on the LDL receptor–deficient background) was reported to increase atherosclerosis via a mechanism attributed to endoplasmic reticulum stress in endothelial cells [32] as well as phenotypic switching in vascular smooth muscle cells [33]. However, the same group also linked the deletion of the AMPKα1 subunit (on the ApoE^-/-^ background) with increased atherosclerotic calcification [34], but decreased atherosclerosis via altered macrophage differentiation [35]. The latter observation, albeit following AMPKα1 subunit deletion, fits best with the observations made during the current investigation and highlights the potential impact of different genetic backgrounds on the physiological consequences of AMPK deletion.

Taken together, the present study revealed that protein expression in bone marrow-derived monocytes from wild-type and AMPKα2^∆MC^ mice was distinct. While the studies focused on the link to methylation, it is clear that other mechanisms can underlie the regulation of many of the proteins found to be differentially regulated. Indeed, in addition to its effects on the maturation of a spectrum of microRNAs [36], and transcription factors [7], the results are consistent with the recent identification of the AMPK, as a modulator of the epigenetic landscape [37]. 

## 4. Materials and Methods

### 4.1. Materials

IL-4, IL-13 and IFN-γ were from PeproTech (Hamburg, Germany). The antibody against MAT2B was from GeneTex/Biozol (GTX115863, 1:1000; Eching, Germany), anti-DNMT1 was from Novus Biologicals/Bio-Techne (Wiesbaden, Germany; NB100-56519; 1:1000). The antibodies against MMP9 (M9570; 1:1000 for Western blot), β-actin (A5541, 1:5000) were from Sigma/Merck (Taufkirchen, Germany). Unless otherwise mentioned, all other materials were from Sigma/Merck or Applichem (Darmstadt, Germany).

### 4.2. Animals

ApoE^-/-^ mice were purchased from Charles River (Sulzfeld, Germany). Floxed AMPKα2 mice were kindly provided by Benoit Viollet (INSERM, U1016, Paris, France) [5] and bred at the Goethe University Hospital animal facility. Floxed AMPKα2 mice were crossed with animals expressing the Cre-recombinase under the control of the *Lysm* promoter (B6.129P2-Lyz2tm1(cre)Ifo/J; Jackson Laboratories) to generate mice lacking the α2 subunit specifically in myeloid cells, as described [18]. The resulting AMPKα2^∆MC^ mice were crossed onto the ApoE^-/-^ background to generate ApoExα2^∆MC^ mice. For the feeding experiments and the partial carotid ligation ApoExα2^fl/fl^xCre^-/-^ (ApoExα2^fl/fl^) and LysM Cre^+/-^ ApoExα2^∆MC^) as controls were used. All animals were housed in conditions that conform to the Guide for the Care and Use of Laboratory Animals published by the United States National Institutes of Health (NIH publication No. 85-23). Both the University Animal Care Committee and the Federal Authority for Animal Research at the Regierungspräsidium Darmstadt (Hessen, Germany) approved the study protocol (#F28/34). For the isolation of organs, mice were killed using 4% isoflurane in air and subsequent exsanguination or decapitation.

### 4.3. Partial Carotid Ligation

ApoExα2^fl/fl^ and ApoExα2^∆MC^ mice underwent partial ligation of the left carotid artery (LCA) as previously described [22], whereas untouched right carotid artery (RCA) and sham LCA were used as controls. Following ligation, the mice were maintained on a cholesterol rich high-fat diet (Ssniff, Soest, Germany) for up to 3 weeks. For the analysis of infiltrated leukocytes, carotid arteries were harvested 1 week after ligation, and for the morphological studies the ligated and non-ligated carotid arteries were isolated after 3 weeks. Carotid arteries were excised, cleaned of connective tissue, pinned on petri dishes coated with agarose and pictures taken using a stereomicroscope (Lumar.V12, Zeiss Jena, Germany) with a 1.5-fold lens. For frozen sections, carotid arteries were embedded together with the aortic arch in Tissue-Tek optimum cutting temperature medium (VWR, Darmstadt, Germany), frozen on dry ice, and stored at −80 °C until used. Three specific locations along the carotid arteries were cryosectioned: distal (close to the aortic arch at the level of the branch point of the right subclavian artery (distal, D), middle (M), and proximal (P) close to the ligation. Frozen sections (5 to 10 µm) were fixed in 4% paraformaldehyde (Roth, Karlsruhe, Germany) for 10 min at room temperature before staining. For Oil red O staining, sections were incubated in Oil red O solution (Sigma-Aldrich/Merck), for 1 h, nuclei staining was performed with Mayer‘s Hämalaun (Roth) and mounted with glycerin. Samples were analyzed using a Zeiss Axio Scope.A1 microscope (Zeiss, Jena, Germany). Plaque area was determined using the Zeiss AxioVs40 V4.8.2.0 software. 

### 4.4. Western Diet 

Six-week-old ApoExα2^fl/fl^ and ApoExα2^∆MC^ mice were fed a western-type diet (Ssniff, Soest, Germany), for 2 or 4 months. After this time, the mice were killed and the vessels were excised, cleaned from connective tissue and either used for dissection to single cells for FACs analysis of infiltrated immune cells or for histological and immunohistochemistry experiments. The excised arteries were pinned on agarose plates, opened and stained with Oil red O after fixation 

To section the aortic root, hearts were excised and fixed overnight in Histofix (4%, Roth) and embedded in Tissue-Tek OCT. Using the cryostat, the aortic roots in the range of the aortic valve were cut into 10 µm sections. Prior to staining, the sections were washed with phosphate buffered saline (PBS) and blocked with 0.3% bovine serum albumin, 5% horse serum (Fisher Scientific) and 0.1% Triton X-100 for 1 h at room temperature. The antibodies against Mac2 (CL8942AP, Cedarlane/Biozol, 1:200, Eching, Germany), MMP9 (M9570, Sigma, 1:200) and Collagen IV (1340-01, SouthernBiotech/Biozol, 1:1000, Eching, Germany) were diluted in PBS and incubated at 4°C overnight. After washing four times with PBS, the secondary antibodies (1:300, donkey anti-rat AB150155, Abcam, Cambridge, Great Britain), or anti-goat Alexa Fluor 568 (1:300, A-11057, Invitrogen/Thermo Fisher Scientific, Frankfurt, Germany) were incubated with FITC conjugated smooth muscle actin antibody (F377, 1:1000, Sigma-Aldrich/Merck). After washing with PBS the sections were embedded in mounting medium: 1 mmol/L dithiothreitol, 50% Glycerin, and 1 µg/mL 4’,6-diamidino-2-phenylindol (DAPI), and the fluorescence was detected using a LSM780 (Zeiss). 

### 4.5. Histochemical Staining

Picrosirius red and Masson Trichome stainings were performed on cryosections washed with PBS. For picrosirius red, slides were incubated for 1 h in 0.1% picrosirius Red solution (Waldeck GmbH, Münster, Germany) in a saturated aqueous solution of picric acid (Sigma/Merck). Sections were destained with 0.05% acetic acid and mounted with Entellan (Sigma/Merck). Manson Trichome staining was performed using a kit according to the manufacturers (Sigma/Merck) protocol. 

### 4.6. RNA Isolation and Real Time Quantitative PCR (RT-qPCR)

Total RNA was extracted using TriReagent (Sigma-Aldrich/Merck) and concentration was determined by OD260nm (Nanodrop, Thermo Scientific, Dreieich, Germany). For the generation of cDNA total RNA (200 ng) was reverse transcribed using the SuperScript III (Invitrogen/Thermo Scientific, Dreieich, Germany) and random hexamer primers according to the manufacturer’s protocol. The amount of mRNA was quantified using the cycle threshold (cT) value using a SYBR green master mix (Biozym, Hessisch Ohlendorf, Germany) with intron spanning primers in a Mic qPCR system (BMS, Australia). CT values obtained were converted into relative amounts based on a standard curve obtained from serial dilutions of a positive control and mRNA levels were normalized to 18S rRNA. Primers for PCR were ordered from Biospring (Frankfurt, Germany), sequences are listed in Table 1.

### 4.7. DNA Methylation Quantification

DNA was isolated using the DNAeasy Blood and Tissue kit from Qiagen (Hilden, Germany). 2 µg of genomic DNA was sheared in ice-cold water (15/15 rounds high/low shear of 10 s on/off cycles) using the Bioruptor plus from Diagenode (Seraing, Belgium) to achieve fragments of 200–1000 bp in length. The immunoprecipitation of methylated DNA was performed using the abcam kit (ab117133, Cambridge, Great Britain) according to the manufacturer’s instructions. In brief, wells were preincubated with an anti-methyl cytosine antibody, following the removal of unbound antibody sheared genomic DNA was added and incubated for 2 h at room temperature. Following several washing steps, the samples were treated with proteinase K and enriched methylated DNA was purified using silica columns. The methylation status of the CpG islands in *Vegfa* and *Mmp9* was assessed by qPCR using the primers listed in Table 2. All samples were normalized to their corresponding input and wild-type animals.

### 4.8. Immunoblotting

Cells were lysed in Triton X-100 buffer and detergent-soluble proteins were solubilized in SDS-PAGE sample buffer, separated by SDS-PAGE and subjected to Western blotting as described [38]. Proteins were visualized by enhanced chemiluminescence using a commercially available kit (Sigma-Aldrich, Merck). 

### 4.9. Fluorescence-activated Cell Sorting

Mice were sacrificed by 4% isoflurane in air, the heart was punctuated and NaCl solution (15 mL 0.9%) containing 5 U/mL heparin was perfused. The carotid arteries or the aortas were excised and cleaned from connective tissue. To obtain single cell suspensions from the entire aortas or carotid arteries the vessels were microdissected and digested with 125 U/mL collagenase type XI, 60 U/mL hyaluronidase type I-s, 60 U/mL DNase1, and 450 U/mL collagenase type I in PBS containing 20 mmol/L HEPES at 37 °C for 1 h [39]. Cell suspension was passed through a 70 μm filter and washed in PBS. For flow cytometric analysis cells were incubated in Fc block solution (CD16/32 1/100 in PBS with 0.1% bovine serum albumin) to prevent non-specific antibody binding. Staining with corresponding antibodies were performed in PBS containing 2% fetal calf serum (FCS; Gibco Life Technologies/Thermo Fisher Scientific, Frankfurt, Germany) and 1 mmol/L EDTA and cells were analyzed with a LSRII/Fortessa flow cytometer (BD Biosciences, Frankfurt, Germany) and analyzed using FlowJo V10. Neutrophils were defined as CD45+, CD11b+ and Gr1+ cells, macrophages were defined as CD45+, CD11b+ and F4/80+ cells. The following antibodies were used: anti-CD3-PE-CF594, anti-CD4-V500, anti-CD11c-AlexaFluor700, anti-CD19-APC-H7, anti-CD326 (EpCAM)-BV711, anti-Ly6C-PerCP-Cy5.5 (BD Biosciences), anti-CD8-eFluor650, anti-CD11b-eFluor605NC (eBioscience, Frankfurt, Germany), anti-CD45-VioBlue, anti-CD49b-PE, anti-MHC-II-APC (Miltenyi Biotec, Bergisch Gladbach, Germany), anti-F4/80-PE-Cy7, and anti-Ly6G-APC-Cy7 (Biolegend/Biozol).

### 4.10. Isolation of Bone Marrow Cells 

Murine bone marrow cells were obtained from 12 weeks old mice. Suspended cells were seeded on tissue culture dishes in RPMI 1640 (Gibco, Life Technologies/Thermo Fisher Scientific, Frankfurt, Germany), and after 30 min the medium was changed to RPMI with 10% FCS. For differentiation to macrophages cells were in the presence of granulocyte macrophage colony-stimulating factor and macrophage colony-stimulating factor (15 ng/mL each; PeproTech, Hamburg, Germany) for 6 days. For the polarization to M1 macrophages the cells were treated for 4 h with LPS (0.1 ng/mL) IFN-γ (1 ng/mL), for the polarization to M2 macrophages IL-4 and IL-14 (each 1 ng/mL) was applied for 16 h. 

### 4.11. Proteomics: Sample Preparation 

Pellets of isolated monocytes were solubilized in 10% SDS, 150 mmol/L NaCl, 100 mmol/L Tris/HCl pH 7.6, 100 mmol/L dithiothreitol. Samples were sonicated for 5 s and heated at 95 °C for 5 min to facilitate protein solubilization. Samples were then incubated at 56 °C for 30 min and centrifuged to remove insoluble material. Total protein (100 µg per sample) was diluted by adding 200 µL, 8 mol/L urea, 50 mmol/L Tris/HCl, pH 8.5 and loaded onto spin filters with a 30 kDa cut off (Microcon, Merck/Millipore, Darmstadt, Germany), and prepared as described [40]. Proteins were digested overnight with trypsin (sequencing grade, Promega, Mannheim, Germany) and eluted peptides were acidified by trifluoroacetic acid to a final concentration of 0.1 % and fractionated on multi-stop-and-go tips (StageTips) containing three strong cation exchange (SCX) disks and a stack of three C18-disks on top. SCX fractionation by StageTips was performed in four steps as described [41]. The first and second fractions were combined and all three fractions of each sample were eluted in wells of microtiter plates. Peptides were dried and resolved in 1% acetonitrile and 0.1% formic acid.

### 4.12. Proteomics: Liquid Chromatography/Mass Spectrometry (LC/MS)

LC/MS was performed using a Q Exactive Plus (Thermo Scientific, Dreieich, Germany) equipped with an ultra-high-performance LC unit (Dionex Ultimate 3000, Thermo Scientific) and a Nanospray Flex Ion-Source (Thermo Scientific). Peptides were loaded on a C18 reversed-phase precolumn followed by separation on a 2.4 μm reprosil C18 resin (Dr. Maisch GmbH, Ammerbuch, Germany) in-house packed picotip emitter tip (diameter 100 μm, 15 cm long from New Objectives, Woburn, USA) using a gradient from eluent A (4% acetonitrile, 0.1% formic acid) to 30% eluent B (80% acetonitrile, 0.1% formic acid) for 110 min followed by a second gradient to 60% eluent B for 45 min with a flow rate 300 nL/minute. MS data were recorded by data dependent acquisition using the Top10 method to select the most abundant precursor ions in positive mode for higher-energy collisional dissociation fragmentation. The lock mass option was enabled to ensure high mass accuracy during many following runs. The full MS scan range was 300 to 2000 *m*/*z* with a resolution of 70,000, and an automatic gain control value of 3 × 10^6^ total ion counts with a maximal ion injection time of 160 ms. Only higher charged ions (2+) were selected for MS/MS scans with a resolution of 17,500, an isolation window of 2 *m*/*z* and an automatic gain control value set to 10^5^ ions with a maximal ion injection time of 150 ms. Selected ions were excluded in a time frame of 30 s following fragmentation event. Fullscan data were acquired in profile and fragments in centroid mode by Xcalibur software (v3.0.63, 27 August 2013, Thermo Fisher Scientific, Waltham, MA, United states, country).

### 4.13. Proteomics: MS Data Analysis 

Xcalibur raw files were analyzed using the Max Quant (1.5.2.8; MPI of Biochemistry, Martinsried, Germany, http://141.61.102.17/maxquant_doku/doku.php?id=start, accessed on: 30 March 2015) proteomics software [42]. The enzyme specificity was set to trypsin, missed cleavages were limited to 2. Acetylation of N-terminus (+42.01) and oxidation of methionine (+15.99) were selected for variable modification, carbamidomethylation (+57.02) on cysteines was set as fixed modification. The mouse reference proteome set from the UniProt Knowledgebase (download June 2015, 76086 entries) was used to identify peptides and proteins and the false discovery rate was set to 5%. Label free quantification values were obtained from at least one identified peptide. For further analysis data were uploaded into Perseus software software (1.5.1.6, http://www.perseus-framework.org/, accessed on: 27 March 2015; MPI for Biochemistry, Martinsried, Germany) [43]. Identifications from the reverse decoy database and known contaminants were excluded. For quantification, proteins were quality filtered according to a minimum of four valid values in at least one experimental group. Missing values were replaced by background values from normal distribution. For statistical comparison, one way ANOVA and subsequent post hoc *t*-tests were used. 

### 4.14. Metabolomics: Liquid Chromatography/Tandem Mass Spectrometry (LC-MS/MS)

The quantification of SAM and SAH was performed by LC-MS/MS. Cells were scraped in acidic methanol (85% methanol, 0.1% formic acid) and snap frozen. The supernatant was used for reversed-phase LC separation using a Waters Acquity HSS T3 column (150 mm × 2.1 mm, 1.8 µm; Waters, Eschborn, Germany) at 35 °C, operated by an Agilent 1290 Infinity pump system (Agilent, Waldbronn, Germany). The LC mobile phase was A) 100% miliQ water with 0.1% formic acid and B) 100% methanol with 0.1% formic acid. Starting condition for the separation was 98% solvent A held for 2 min, followed by a 0.5 min gradient from 60% A to 5% A, hold 55 for 0.5 min, followed by an equilibration step to 98% A, making 6 min total LC run time. The flow rate was 330 μL/minute, injection volume 5 μL. The QTrap 5500 mass spectrometer (Sciex, Darmstadt, Germany) was operated using positive mode electrospray ionisation using the following parameter: CUR 30 psi, CAD medium, IS 4000 V, TEM 350 °C, GS1 15 psi, GS2 20 psi. Two MRM transitions were used for each metabolite, one serving as quantifier one as qualifier, respectively (SAH: *m*/*z* 385.1/136.1, 385.1/88.0; SAM: *m*/*z* 399.1/250.1, 399.1/136.1). Calibration curves were performed with authentic standards. The intensity of the measured metabolite was normalized to SAM-d3 internal standard. Analyst 1.6.2 and MultiQuant 3.0 (Sciex, Darmstadt, Germany), were used for data acquisition and analysis, respectively.

### 4.15. Statistical Analysis

Data are expressed as mean ± SEM. Statistical evaluation was performed using Student’s t test for unpaired data, one-way ANOVA followed by a Bonferroni t test or ANOVA for repeated measures where appropriate. Values of *p* < 0.05 were considered statistically significant. 

## Figures and Tables

**Table 1:** Primers used for RT-qPCR.

Target mRNA	Forward Primer	Reverse Primer
18S RNA	5′-CTTTGGTCGCTCGCTCCTC-3′	5′-CTGACCGGGTTGGTTTTGAT-3′
VEGF	5′-GCACTGGACCCTGGCTTTACTGCTGTA-3′	5′-GAACTTGATCACTTCATGGGACTTCTGCTC-3′
MMP9	5′-GAAGGCAAACCCTGTGTT-3′	5′-AGAGTACTGCTTGCCCAGGA-3′
Mat2B	5′-CAGAGGTTCCCCACACATGT-3′	5′-GGGGAGGTTGAAGGCATCTG-3′
DNMT1	5′-AAGGGGGCCCTGACCGCTTC-3′	5′-CCGAAATGCCTGGGCTGCCG-3′
IL-1β	5′-CAGGCAGGCAGTATCACTCA-3′	5′-AGCTCATATGGGTCCGACAG-3′
TNF-α	5′-GGCCTTCCTACCTTCAGACC-3′	5′-CCGGCCTTCCAAATAAATAC-3′
iNOS	5′-GTGGTGACAAGCACATTTGG-3′	5′-GGCCTTCCTACCTTCAGACC-3′
Arginase	5′-GTGAAGAACCCACGGTCTGT-3′	5′-CTGGTTGTCAGGGGAGTGTT-3′
FIZZ1	5′-CCCTTCTCATCTGCATCTCC-3′	5′-CAGTAGCAGTCATCCCAGCA-3′
YM1	5′-CTGGAATTGGTGCCCCTACAA-3′	5′-TCATAACCAACCCACTCATTACC-3′

**Table 2:** Primers used for DNA methylation quantification.

Target Region	Forward Primer	Reverse Primer
*Vegfa*	5′-CTCCTCTCCCTTCTGGAACC-3′	5′-GAGGGGAGGAAGAGAAGGAA-3′
*Mmp9*	5′-CAATCCCTAGTCGCTGCTTC-3′	5′-AGGAAGGGACTCAATCAGCA-3′

## Supplementary Materials

Supplementary materials can be found at https://www.mdpi.com/1422-0067/20/12/3005/s1.

## Abbreviations

AMPK	AMP-activated protein kinase
AMP	Adenosine monophosphate
ApoE	Apolipoprotein E
ATP	Adenosine triphosphate
CpG	Cytosine-guanine dinucleotide
DNMT	DNA methyltransferase
FACs	Fluorescence-activated cell sorting
FIZZ1	Found in inflammatory zone 1
IFN	Interferon
IL	Interleukin
iNOS	inducible nitric oxide synthase
LCA	Left carotid artery
LC/MS	Liquid chromatography/Mass spectrometery
LPS	Lipopolysaccharide
MAT2B	Methionine adenosyltransferase 2B
MMP	Matrix metalloproteinase
RCA	Right carotid artery
SAM	S-adenosyl methionine
SAH	S-adenosyl homocysteine
TNFα	Tumor necrosis factor alpha
VEGF	Vascular endothelial cell growth factor
YM1	Chitinase 3 like protein 3

## References

[1] Ross F.A., MacKintosh C., Hardie D.G. (2016). AMP-activated protein kinase: A cellular energy sensor that comes in 12 flavours. FEBS J..

[2] Hardie D.G., Lin S.C. (2017). AMP-activated protein kinase-not just an energy sensor. F1000Research.

[3] Foretz M., Guihard S., Leclerc J., Fauveau V., Couty J.P., Andris F., Gaudry M., Andreelli F., Vaulont S., Viollet B. (2010). Maintenance of red blood cell integrity by AMP-activated protein kinase α1 catalytic subunit. FEBS Lett..

[4] Wang S., Dale G.L., Song P., Viollet B., Zou M.H. (2010). AMPKα1 deletion shortens erythrocyte life span in mice. J. Biol. Chem..

[5] Viollet B., Andreelli F., Jorgensen S.B., Perrin C., Geloen A., Flamez D., Mu J., Lenzner C., Baud O., Bennoun M. (2003). The AMP-activated protein kinase α2 catalytic subunit controls whole-body insulin sensitivity. J. Clin. Investig..

[6] Viollet B., Andreelli F., Jorgensen S.B., Perrin C., Flamez D., Mu J., Wojtaszewski J.F., Schuit F.C., Birnbaum M., Richter E. (2003). Physiological role of AMP-activated protein kinase (AMPK): Insights from knockout mouse models. Biochem. Soc. Trans..

[7] Salt I.P., Hardie D.G. (2017). AMP-Activated protein kinase: An ubiquitous signaling pathway with key roles in the cardiovascular system. Circ. Res..

[8] Morrow V.A., Foufelle F., Connell J.M.C., Petrie J.R., Gould G.W., Salt I.P. (2003). Direct activation of AMP-activated protein kinase stimulates nitric oxide synthesis in human aortic endothelial cells. J. Biol. Chem..

[9] Stahmann N., Woods A., Spengler K., Heslegrave A., Bauer R., Krause S., Viollet B., Carling D., Heller R. (2010). Activation of AMP-activated protein kinase by vascular endothelial growth factor mediates endothelial angiogenesis independent of nitric-oxide synthase. J. Biol. Chem..

[10] Liu C., Liang B., Wang Q., Wu J., Zou M.H. (2010). Activation of the AMP-activated protein kinase α1 alleviates endothelial cell apoptosis by increasing the expression of anti-apoptotic proteins BCL-2 and survivin. J. Biol. Chem..

[11] Zippel N., Abdel Malik R., Frömel T., Popp R., Bess E., Strilic B., Wettschureck N., Fleming I., Fisslthaler B. (2013). Transforming growth factor-β-activated kinase 1 regulates angiogenesis via AMP-activated protein kinase-α1 and redox balance in endothelial cells. Arterioscler. Thromb. Vasc. Biol..

[12] Wang S., Zhang M., Liang B., Xu J., Xie Z., Liu C., Viollet B., Yan D., Zou M.H. (2010). AMPKa2 deletion causes aberrant expression and activation of NAD(P)H oxidase and consequent endothelial dysfunction in vivo. Role of 26S proteasomes. Circ. Res..

[13] Bess E., Fisslthaler B., Frömel T., Fleming I. (2011). Nitric oxide-induced activation of the AMP-activated protein kinase α2 subunit attenuates IκB kinase activity and inflammatory responses in endothelial cells. PLoS ONE.

[14] Carroll K.C., Viollet B., Suttles J. (2013). AMPKα1 deficiency amplifies proinflammatory myeloid APC activity and CD40 signaling. J. Leukoc. Biol..

[15] Sag D., Carling D., Stout R.D., Suttles J. (2008). Adenosine 5-monophosphate-activated protein kinase promotes macrophage polarization to an anti-inflammatory functional phenotype. J. Immunol..

[16] Zhu Y.P., Brown J.R., Sag D., Zhang L., Suttles J. (2015). Adenosine 5-monophosphate-activated protein kinase regulates IL-10-mediated anti-inflammatory signaling pathways in macrophages. J. Immunol..

[17] Kohlstedt K., Trouvain C., Namgaladze D., Fleming I. (2011). Adipocyte-derived lipids increase angiotensin-converting enzyme (ACE) expression and modulate macrophage phenotype. Basic Res. Cardiol..

[18] Abdel Malik R., Zippel N., Frömel T., Heidler J., Zukunft S., Walzog B., Ansari N., Pampaloni F., Wingert S., Rieger M.A. (2017). AMP-activated protein kinase α2 in neutrophils regulates hypoxia-inducible factor-1α and a network of proteins affecting metabolism and vascular repair after ischemia. Circ. Res..

[19] Igarashi K., Katoh Y. (2013). Metabolic aspects of epigenome: Coupling of S-adenosylmethionine synthesis and gene regulation on chromatin by SAMIT module. Epigenetics Dev. Dis..

[20] Carling D., Clarke P.R., Zammit V.A., Hardie D.G. (1989). Purification and characterization of the AMP-activated protein kinase. Copurification of acetyl-CoA carboxylase kinase and 3-hydroxy-3-methylglutaryl-CoA reductase kinase activities. Eur. J. Biochem..

[21] Fisslthaler B., Fleming I., Keserü B., Walsh K., Busse R. (2007). Fluid shear stress and NO decrease the activity of the hydroxy-methylglutaryl coenzyme A reductase in endothelial cells via the AMP-activated protein kinase and FoxO1. Circ. Res..

[22] Nam D., Ni C.W., Rezvan A., Suo J., Budzyn K., Llanos A., Harrison D., Giddens D., Jo H. (2009). Partial carotid ligation is a model of acutely induced disturbed flow, leading to rapid endothelial dysfunction and atherosclerosis. Am. J. Physiol. Heart Circ. Physiol..

[23] Cui S., Li W., Lv X., Wang P., Gao Y., Huang G. (2017). Folic acid supplementation delays atherosclerotic lesion development by modulating *Mcp1* and *Vegf* DNA methylation levels in vivo and in vitro. Int. J. Mol. Sci..

[24] Marin T.L., Gongol B., Zhang F., Martin M., Johnson D.A., Xiao H., Wang Y., Subramaniam S., Chien S., Shyy J.Y. (2017). AMPK promotes mitochondrial biogenesis and function by phosphorylating the epigenetic factors DNMT1, RBBP7, and HAT1. Sci. Signal.

[25] Palomer X., Salvado L., Barroso E., Vazquez-Carrera M. (2013). An overview of the crosstalk between inflammatory processes and metabolic dysregulation during diabetic cardiomyopathy. Int. J. Cardiol..

[26] Ouimet M., Ediriweera H.N., Gundra U.M., Sheedy F.J., Ramkhelawon B., Hutchison S.B., Rinehold K., van Solingen S.C., Fullerton M.D., Cecchini K. (2015). MicroRNA-33-dependent regulation of macrophage metabolism directs immune cell polarization in atherosclerosis. J. Clin. Investig..

[27] Price N.L., Rotllan N., Canfran-Duque A., Zhang X., Pati P., Arias N., Moen J., Mayr M., Ford D.A., Baldan A. (2017). Genetic dissection of the impact of miR-33a and miR-33b during the progression of atherosclerosis. Cell Rep..

[28] Ohki Y., Heissig B., Sato Y., Akiyama H., Zhu Z., Hicklin D.J., Shimada K., Ogawa H., Daida H., Hattori K. (2005). Granulocyte colony-stimulating factor promotes neovascularization by releasing vascular endothelial growth factor from neutrophils. FASEB J..

[29] Döring Y., Drechsler M., Wantha S., Kemmerich K., Lievens D., Vijayan S., Gallo R.L., Weber C., Soehnlein O. (2012). Lack of neutrophil-derived CRAMP reduces atherosclerosis in mice. Circ. Res..

[30] Wantha S., Alard J.E., Megens R.T.A., van der Does A.M., Döring Y., Drechsler M., Pham C.T.N., Wang M.W., Wang J.M., Gallo R.L. (2013). Neutrophil-derived cathelicidin promotes adhesion of classical monocytes. Circ. Res..

[31] Zhu H., Zhang M., Liu Z., Xing J., Moriasi C., Dai X., Zou M.H. (2016). AMP-Activated protein kinase α1 in macrophages promotes collateral remodeling and arteriogenesis in mice in vivo. Arterioscler. Thromb. Vasc. Biol..

[32] Dong Y., Zhang M., Liang B., Xie Z., Zhao Z., Asfa S., Choi H.C., Zou M.H. (2010). Reduction of AMP-activated protein kinase α2 increases endoplasmic reticulum stress and atherosclerosis in vivo. Circulation.

[33] Ding Y., Zhang M., Zhang W., Lu Q., Cai Z., Song P., Okon I.S., Xiao L., Zou M.H. (2016). AMP-Activated protein kinase alpha 2 deletion induces VSMC phenotypic switching and reduces features of atherosclerotic plaque stability. Circ. Res..

[34] Cai Z., Ding Y., Zhang M., Lu Q., Wu S., Zhu H., Song P., Zou M.H. (2016). Ablation of adenosine monophosphate-activated protein kinase α1 in vascular smooth muscle cells promotes diet-induced atherosclerotic calcification in vivo. Circ. Res..

[35] Zhang M., Zhu H., Ding Y., Liu Z., Cai Z., Zou M.H. (2017). AMP-activated protein kinase α1 promotes atherogenesis by increasing monocyte-to-macrophage differentiation. J. Biol. Chem..

[36] Kohlstedt K., Trouvain C., Boettger T., Shi L., Fisslthaler B., Fleming I. (2013). AMP-activated protein kinase regulates endothelial cell angiotensin-converting enzyme expression via p53 and the post-transcriptional regulation of microRNA-143/145. Circ. Res..

[37] Gongol B., Sari I., Bryant T., Rosete G., Marin T. (2018). AMPK: An epigenetic landscape modulator. Int. J. Mol. Sci..

[38] Fleming I., Fisslthaler B., Dixit M., Busse R. (2005). Role of PECAM-1 in the shear-stress-induced activation of Akt and the endothelial nitric oxide synthase (eNOS) in endothelial cells. J. Cell Sci..

[39] Galkina E., Kadl A., Sanders J., Varughese D., Sarembock I.J., Ley K. (2006). Lymphocyte recruitment into the aortic wall before and during development of atherosclerosis is partially L-selectin dependent. J. Exp. Med..

[40] Wisniewski J.R., Zougman A., Nagaraj N., Mann M. (2009). Universal sample preparation method for proteome analysis. Nat. Methods.

[41] Rappsilber J., Mann M., Ishihama Y. (2007). Protocol for micro-purification, enrichment, pre-fractionation and storage of peptides for proteomics using StageTips. Nat. Protoc..

[42] Tyanova S., Temu T., Cox J. (2016). The MaxQuant computational platform for mass spectrometry-based shotgun proteomics. Nat. Protoc..

[43] Tyanova S., Temu T., Sinitcyn P., Carlson A., Hein M.Y., Geiger T., Mann M., Cox J. (2016). The Perseus computational platform for comprehensive analysis of (prote)omics data. Nat. Methods.

